# Testes Proteases Expression and Hybrid Male Sterility Between Subspecies of *Drosophila pseudoobscura*

**DOI:** 10.1534/g3.119.300580

**Published:** 2019-02-05

**Authors:** Doaa Alhazmi, Seth Kaleb Fudyk, Alberto Civetta

**Affiliations:** Department of Biology, University of Winnipeg, MB, Canada, R3B 2E9

**Keywords:** Speciation, *Drosophila*, proteases expression, gene interactions, cis-regulation

## Abstract

Hybrid male sterility (HMS) is a form of postmating postzygotic isolation among closely related species that can act as an effective barrier to gene flow. The Dobzhansky-Muller model provides a framework to explain how gene interactions can cause HMS between species. Genomics highlights the preponderance of non-coding DNA targets that could be involved in gene interactions resulting in gene expression changes and the establishment of isolating barriers. However, we have limited knowledge of changes in gene expression associated with HMS, gene interacting partners linked to HMS, and whether substitutions in DNA regulatory regions (*cis*) causes misexpression (*i.e.*, expression of genes beyond levels found in parental species) of HMS genes in sterile hybrids. A previous transcriptome survey in a pair of *D. pseudoobscura* species found male reproductive tract (MRT) proteases as the largest class of genes misregulated in sterile hybrids. Here we assay gene expression in backcross (BC) and introgression (IG) progeny, along with site of expression within the MRT, to identify misexpression of proteases that might directly contribute to HMS. We find limited evidence of an accumulation of *cis*-regulatory changes upstream of such candidate HMS genes. The expression of four genes was differentially modulated by alleles of the previously characterized HMS gene *Ovd*.

In speciation, prezygotic and postzygotic barriers evolve that isolate divergent populations. Many studies on the genetic basis of speciation have focused on postmating postzygotic (*i.e.*, sterility/ inviability of hybrids) reproductive isolation and some general patterns have emerged from these studies. Commonly, there is a disproportionate contribution of the sex chromosome to heterogametic F_1_ inviability/sterility (the “large-X effect”) but gene interactions are needed for full establishment of isolation barriers (Coyne and Orr 1989; Masly and Presgraves 2007; Presgraves 2008; Cattani and Presgraves 2012; Dufresnes *et al.* 2016). The important role of gene × gene interactions in the onset of HMS is well illustrated in the Dobzhansky-Muller (DM) model of incompatibilities (Dobzhansky 1937; Muller 1942). The model draws attention to the relationship between populations’ divergence and the accumulation of incompatible allele interactions leading to phenotypes such as HMS. Several studies have mapped loci that contribute to the onset of HMS and a few single major HMS genes have been identified. For example, in *Drosophila*, Odysseus-site homeobox (*OdsH*) (Ting *et al.* 1998), *JYAlpha* (Masly *et al.* 2006), and Overdrive (*Ovd*) (Phadnis and Orr 2009); in mouse *Prdm9* (Mihola *et al.* 2009). Many empirical studies have also highlighted that gene interactions are crucial for the manifestation of HMS, and in many cases the effectiveness of the aforementioned major HMS genes has been shown to be dependent on genetic background (Perez and Wu 1995; Orr and Irving 2001; Presgraves *et al.* 2003; Tao *et al.* 2003; Sawamura *et al.* 2004; Chang and Noor 2010; Phadnis 2011).

The eukaryotic genome consists mostly of non-coding genetic elements, approximately 80% of the *D. melanogaster* euchromatic genome is noncoding (Halligan and Keightley 2006), creating many opportunities for interactions that involve different DNA targets, like promoter elements, enhancers, and silencers, that can affect gene expression. Despite the preponderance of non-coding DNA targets potentially affecting gene expression and the well-acknowledged importance of gene interactions in the onset of HMS, we lack knowledge of what changes in gene expression can trigger HMS, gene interacting partners or networks involved, and whether substitutions in DNA regulatory regions (*cis*) and/or proteins (*trans*) modulate gene misexpression in sterile hybrids.

Recent reviews have summarized how changes in gene expression could impact hybrid phenotypes (Civetta 2016; Mack and Nachman 2017). In *Drosophila*, the use of backcross progeny with fertile partial hybrids controls as well as species pairs in which F1 hybrids follow Darwin’s corollary to Haldane’s rule (*i.e.*, unidirectional sterility) have helped identify a handful of misregulated spermatogenesis genes linked to sterility (Michalak and Noor 2004; Ma and Michalak 2011; Gomes and Civetta 2014). However, there has been a general lack of studies that utilized RNA sequencing technologies to investigate genome-wide patterns of misexpression in sterile males without focusing on candidate gene assays. Taking advantage of a *Drosophila* species pair showing unidirectional male sterility, more genes were found to be misregulated in the sterile F1 male hybrids than in fertile F1 males (Gomes and Civetta 2015). Gene ontology analysis identified proteases as the largest class among genes uniquely misexpressed in the MRT of sterile F1 hybrids, with four genes located within a previously identified HMS locus (Gomes and Civetta 2015). Proteolytic genes are interesting as they have been associated with male sterility and impair fertilization capacity in a wide variety of organisms (Friedländer *et al.* 2001; Subirán *et al.* 2010; LaFlamme *et al.* 2012; Zhao *et al.* 2012; Bosler *et al.* 2014), but their role in HMS and speciation is largely unexplored. Another RNA sequencing survey of genome-wide expression in species of mice where HMS is unidirectional identified genes with roles in cell-cycle control and highly expressed in testis as primary candidates for the establishment of reproductive incompatibilities between the species assayed (Mack *et al.* 2016). Finally, a study of a species pair of Hawaiian *Drosophila* using backcross (BC) males with similar genomic background but different sperm phenotypes identified genes misregulated in spermless BC males that phenotypically resembled F1 sterile male hybrids, without overrepresentation of any functionally annotated gene class (Brill *et al.* 2016). Transcriptome surveys are useful to identify, based on expression changes associated with the sterile condition, divergence in gene regulation that might cause hybrid incompatibilities. However, reciprocal F1 hybrid males are not fully equivalent in their genome composition as they experience different X-autosome and maternal-nuclear interactions. We lack knowledge on how asymmetries between F1 hybrids in X chromosome, cytoplasm and maternal effects could differentially affect gene expression in F1 hybrids irrespectively of their fertility/sterility condition.

Differences in gene expression between species can be driven by sequence divergence that is linked to the affected genes (*cis*) or by divergence in unlinked diffusible products, such as proteins (*trans*). In comparisons between *D. p. pseudoobscura* and *D. p. bogotana*, we have found a preponderance of *cis* rather than *trans* genome-wide regulatory divergence between species (Gomes and Civetta 2015), which agrees with findings using other pairs of species (Wittkopp *et al.* 2008; Emerson and Li 2010; Gordon and Ruvinsky 2012; Coolon *et al.* 2014; Metzger *et al.* 2017; Nourmohammad *et al.* 2017). However, a greater genome-wide contribution of *cis*-regulatory differences to divergence in gene expression between species does not warrant that the same is true for speciation genes. Given the observation of fast evolution of sex chromosome protein coding genes, and examples of X-linked mapped HMS proteins with putative DNA binding domains (Bayes and Malik 2009; Phadnis and Orr 2009; Davies *et al.* 2016) it is possible for HMS species-specific proteins to differentially modulate target genes expression.

The misregulation of proteases previously identified in a genome wide screen (Gomes and Civetta 2015) could directly affect fertility, or do so indirectly by interacting with alleles at other loci. Here we use a combination of backcross genetics and gene expression assays to identify ten proteases that when misexpressed might directly contribute to F1 HMS. Among these ten proteases, seven are prioritized as candidate HMS genes based on patterns of tissue expression within the MRT. We also identify four proteases whose misexpression is affected by the allele status of the *D. pseudoobscura* previously identified major X-linked HMS gene *Ovd* (Phadnis and Orr 2009). The use sequence data analysis shows, with the exception of one gene, no evidence of enrichment in *cis*-regulatory divergence fueling gene-specific misexpression of HMS gene candidates.

## Materials and Methods

### Drosophila stocks and crosses

All flies were maintained on cornmeal–molasses–yeast–agar (CMYA) medium at constant temperature on a 12-hour light–dark cycle. Virgin females were collected post-eclosion and flies were mass crossed in bottles containing CMYA medium. All *Drosophila* stocks were purchased from the *Drosophila* Species Stock Centre (https://stockcenter.ucsd.edu/). We used a wild-type *D. p. pseudoobscura* (14011–0121.139) and a wild-type *D. p. bogotana* (14011-0121.175) strain as well as two *D. p. pseudoobscura* mutant strains. One of the mutant stocks carries an X-linked yellow (*y*) body coloration mutation (14011-0121.06) while the other strain (14011-0121.08) carries, among others, an X-linked sepia (*se*) eye color mutation.

**Figure 1:**
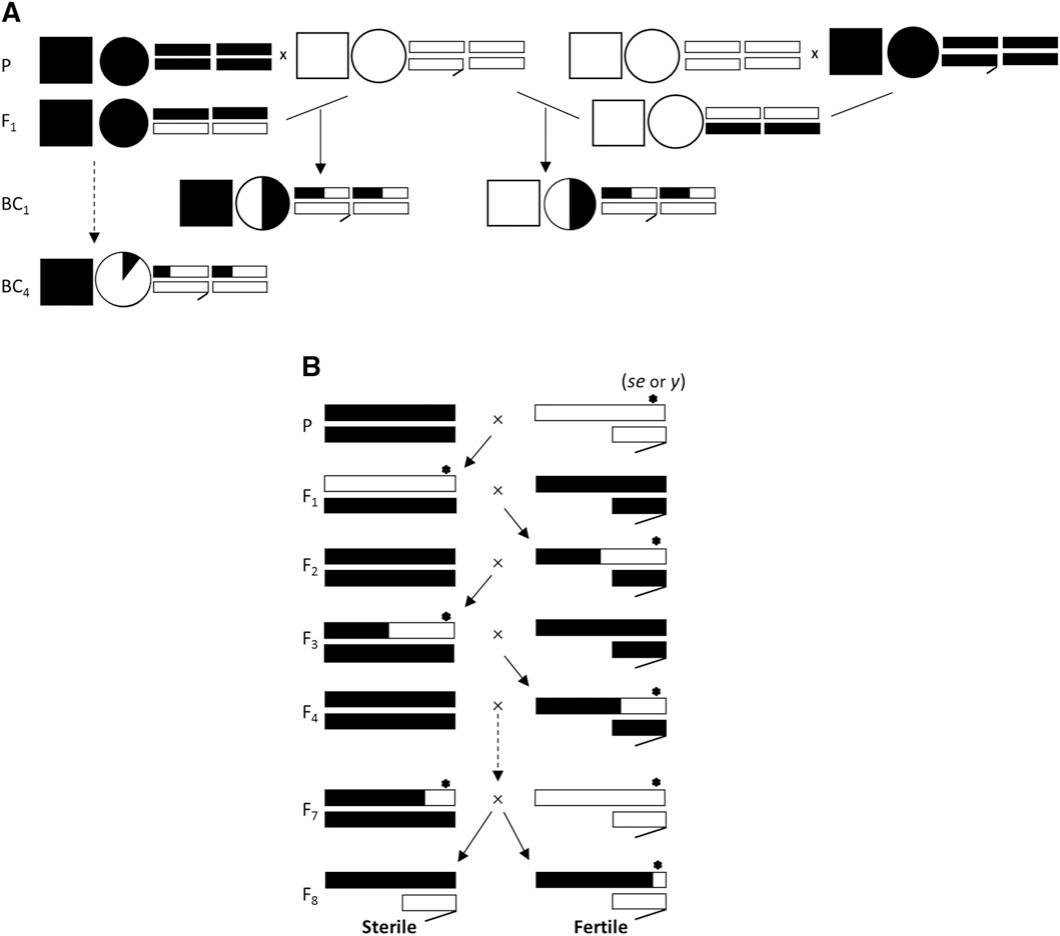
Backcross males (A) and introgression design used to swap fertility/sterility alleles (B). Mitochondrial genome and cytoplasmic factors are represented as squares and circles respectively. Sex chromosomes and autosomes are shown as rectangles. The origin and approximate proportion of each parental genome content is shown by black (*D. p. bogotana*) and white (*D. p. pseudoobscura*) fillings. In the crossing scheme used to replace an X-linked HMS allele with a fertile allele, a visible yellow body color marker (*y*) and a sepia eyes mutation (*se*: linked to *Ovd*) were used to track allele segregation.

First (BC1_b_) and fourth (BC4) generation backcross males were created independently to sample different types of maternal-nuclear and X-autosomal interactions by crossing wild-type *D. p. bogotana* females to *D. p. pseudoobscura* males and then backcrossing the fertile F1 females to *D. p. pseudoobscura* males. The BC4 males were obtained by backcrossing fertile BC1_b_ females to *D. p. pseudoobscura* males. BC1_p_ males were generated by crossing wild-type *D. p. pseudoobscura* females to *D. p. bogotana* males and then backcrossing the fertile F1 females to *D. p. pseudoobscura* males (Figure 1A). A different backcross design was used to replace *D. p. bogotana* X-linked sterility alleles with the corresponding fertile *D. p. pseudoobscura* alleles in males with an otherwise genome composition similar to sterile F1 male hybrids (Figure 1B). For this, we took advantage of the fact that two X-linked sterility loci map near both *y* and *se* mutations (Phadnis and Orr 2009, Phadnis 2011). All backcrosses were created at different times and they represent independent events of recombination.

The average fecundity of the BC and IG males was assayed following a procedure described elsewhere (Gomes and Civetta 2014). Briefly, five seven-day-old focal males (*i.e.*, BC1, BC4 and F8 males – Figure 1) were housed with five *D. p. bogotana* virgin females of the same age. Each cross was replicated six times. Flies were left together for 48 hr in vials (vials 1 to 6) containing CMYA food supplemented with yeast. After 48 hr, males were removed and five days after the original setup, females were transferred to a new vial for an additional 5 days. Fecundity was assayed as the total number of offspring (adult flies) produced from each vial. Counts were done for up to 24 days after the initial set-up to avoid overlapping generations. The same protocol was used to assay parental species male fecundity by using females and males of the same species.

### Gene expression

We extracted total RNA from the entire MRT of 7 days-old focal males as well as parental wild-type strains. We also performed extractions of total RNA from testes (T), seminal vesicles (SV), accessory glands (AG) and the ejaculatory bulb (EB) of same age *D. p. pseudoobscura* males.

The Bio-Rad Aurum total RNA mini kit was used for RNA extractions. In all experiments, at least three biological replicates were obtained from parental species and focal males, each sample consisting of tissue from 10-15 males. RNA samples were quantified and tested for quality using a Nanodrop (Thermo Fisher Scientic). cDNA was synthesized from equivalent amounts of total RNA using the iScript cDNA synthesis kit (Bio-Rad). Primers were designed using Primer3Plus (http://www.primer3plus.com/) with the product size ranging from 75 to 85 base pairs long (Table S1). When possible, intron-spanning primers were used to monitor any genomic DNA contamination. We targeted 19 proteases previously identified as uniquely misregulated in the sterile F1 hybrid males resulting from crosses between *D. p. bogotana* females and *D. p. pseudoobscura* males (Gomes and Civetta 2015). Gene expression was quantified using the BioRad CFX96 Real-Time PCR Detection System. The reactions were performed using the IQ SYBR Green quantitative real-time PCR kit (Bio-Rad). In all assays, two reference genes (RpL32 and RpS18) were used to normalize RT-PCR results for each target gene. Cycling conditions for RT-PCR were the same for all genes: an initial denaturation step for 5 min at 95°, followed by 35 cycles of 95° for 45 sec, 58° for 30 sec and 72° for 45 sec. The effciency of all primers was tested by creating a standard curve of threshold cycle values from a template dilution series. Gene expression was determined by calculating ∆Cq as the Cq of the reference gene (RpL32 or RpS18) minus the Cq value of the target gene. Data were analyzed using (ANOVA) to test the null hypothesis that genes mean relative expression among crosses is not different. If significant differences were detected among crosses, a Scheffe’s post-hoc test was used to determine statistically significant differences between samples. FDR corrected q-values were used for all tests of significance.

### Identification of species sequence divergence in putative cis-regulatory regions

Genomic DNA was extracted from pools of 5 flies and genes PCR amplified. PCR products were cleaned using a Bio-Tek E.Z.N.A Cycle Pure Kit (Omega) and their concentration measured using a Nanodrop spectrophotometer. Cleaned PCR products were sent for Sanger sequencing to the Centre for Applied Genomics (TCAG) at the Hospital for Sick Children in Toronto. We sequenced *D. p. pseudoobscura* (14011–0121.139) and *D. p. bogotana* (14011–0121.175) seven candidate HMS genes derived from our genetics and gene expression assays. Approximately 1Kb upstream, as well as a few hundred bases downstream of the transcription start site (TSS), was aligned using MUSCLE within MEGA to the currently available *D. p. pseudobscura* gene sequences in FlyBase (https://flybase.org/). Fixed nucleotide substitutions between species can be overestimated if polymorphism data are not considered. We checked whether putative fixed changes could possibly be shared polymorphism by BLASTn of our sequences to the sequence reads of 43 *D. p. pseudoobscura* strains (Fuller *et al.* 2014) available at the Short Read Archive (SRA) at the National Center for Biotechnology Information (NCBI). We retained BLAStn matches with higher than 90% identity to the query. The location of fixed substitutions between species was mapped relative to the transcription start site (TSS) (Ohler *et al.* 2002; Roy and Singer 2015). We look for evidence of accumulation of substitutions in *cis*-regulatory regions by using an empirical cumulative distribution function. For any gene region, a monotonic increase in substitutions (n) was identified by calculating G, a measure of the difference between the relative occurrence of a nucleotide change and its relative position in the sequence (Tang and Lewontin 1999; Civetta *et al.* 2016). Differences between the values of G between any two events (ΔG) measure the differential accumulation of nucleotide substitutions. We reject uniform distribution of substitutions if the ΔG with the highest absolute value (T) is higher than T*, where T* is a null random distribution of T obtained using Monte Carlo simulations to produce 100,000 samples of n events by sampling without replacement along a sequence of length N (Tang and Lewontin 1999). Because our *a priori* hypothesis is that there should be an accumulation of substitutions in *cis*-regulatory regions (hotspots) for misregulated genes, we performed one-tailed tests looking for sequence regions where ΔG is positive. A source code of the program used to implement the analysis (Civetta *et al.* 2016) is available in the figshare data repository (Supplementary material).

### Data availability

Supplementary tables, source code for sequence data analysis and raw data for gene expression and fecundity assays have been submitted to the figshare repository. Gene sequences have been deposited in GenBank under accession numbers MK370073 to MK370086 and sequence alignments are available in the Figshare repository. Supplemental material available at Figshare: https://doi.org/10.25387/g3.7560578.

## Results

### Misregulation of proteases in fertile BC and IG male progeny

All BC and IG males were generated through independently setup crosses and therefore represent different events of genomic recombination. Both BC1 and BC4 males were fully fertile. The presence of either *D. p. bogotana* or *D. p. pseudoobscura* alleles at the *y* locus in F8 IG males made no difference in terms of fecundity (Table 1). We suspect this is likely a consequence of *y* not being tightly linked to a previously mapped X-linked sterility QTL (Phadnis 2011). The introgression of a *D. p. bogotana se+* allele caused the expected result of sterility with the *D. p. pseudoobscura se* allele restoring fertility (Table 1).

**Table 1:** Mean fecundity and standard error (SE) of parental species, backcross and introgression males. Reciprocal BC1 males are from *D. p. bogotana* (BC1_b_) and *D. p. pseudoobscura* (BC1_p_) parental females. BC4 males are from a fourth-generation backcross (Figure 1A). Introgression males were generated as shown in figure 1B and they are either yellow (F8*^y^*) *vs.* non-yellow (F8*^y^*^+^) body or sepia (F8*^se^*) *vs.* non-sepia (F8*^se^*^+^) eyes males

Male	Mean Fecundity (SE)
***D. p. pseudoobscura***	90.3 (4.2)
***D. p. bogotana***	83.7 (4.2)
**BC1_b_**	100.5 (1.7)
**BC1_p_**	116.3 (5.3)
**BC4**	97.5 (3.1)
**F8*^y^***	117.2 (17.4)
**F8*^y^*^+^**	104.5 (22.7)
**F8*^se^***	107.7 (2.7)
**F8*^se^*^+^**	0.0 (0.0)

**Figure 2:**
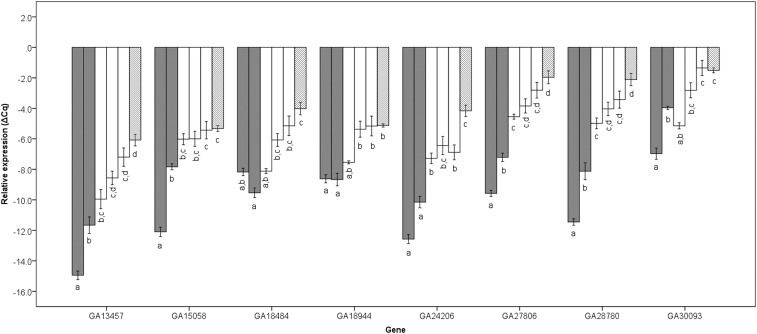
Mean relative expression and standard error of genes in backcross males, that are misregulated relative to parental species, compared to the expression in sterile F1 male hybrids. Parental species are shown in gray, BC progeny in white and sterile F1 males are hatched. For each gene, from left to right, *D. p. bogotana*, *D. p. pseudoobscura*, BC_4_, BC_1p_ (from parental *D. p. pseudoobscura* females), BC_1b_ (from parental *D. p. bogotana* females) and sterile F1 hybrid males. Shared letters identify non-significantly different groups (post-hoc Scheffe’s test, FDR corrected q< 0.05).

Comparisons of relative gene expression showed significant gene and reference gene effects (ANOVA: F_18,1204_= 141.6, *P* < 0.001 and F_1,1204_= 100.3, *P* < 0.001 respectively), but no gene × reference gene effect interaction (F_18,1204_= 0.03, *P* = 1.0) as relative gene expression (∆Cq) was always higher when using RpS18 as a reference. Eight genes (GA13457, GA15058, GA18484, GA18944, GA24206, GA27806, GA28780 and GA30093) were found to be misregulated between fertile BC males and parental (*D. p. pseudoobscura* and *D. p. bogotana*) species (Scheffe’s post-hoc test; Table 2). The level of misexpression detected in fertile BC male progeny could reflect quantitative differences that are not equivalent to misregulation in sterile F1 males. We found that in all cases of misexpression in fertile BC progeny, at least one of the fertile BC progeny was not significantly different from sterile F1 males (Scheffe’s post-hoc test; Figure 2) indicating that the misregulation of these genes is not directly associated with F1 HMS. BC_1b_ males have the same mitochondrial composition as sterile F1 males but a hybrid cytoplasmic and nuclear content that is different from sterile F1 males. The reciprocal BC1_p_ fertile males are on average identical to BC1_b_ males, except for their mitochondrial genome (Figure 1A). The fact that all genes were not differentially expressed between BC1_b_ and reciprocal BC1_p_ males (Figure 2) shows that the misregulation of these genes is not driven by mitochondrial-nuclear interaction. Only three genes were misregulated in BC4 fertile males relative to parental species, GA24206, GA27806 and GA28780 (Figure 2). BC4 males have only an average 3–4% content of *D. p. bogotana* genome in a *D. p. pseudoobscura* background, thus limiting opportunities for interspecies interactions that might affect gene targets expression. The fact that GA24206, GA27806 and GA28780 were misregulated in BC4 males suggests that few *D. p. bogotana* alleles are sufficient to trigger misregulation of these targets.

**Table 2:** Mean relative expression for all genes assayed in parental species, backcross and X-allele introgressions. Gene expression values in fertile backcross and introgression progeny significantly different from both parental species are shown bolded and marked with an asterisk (post-hoc Scheffe’s test, FDR corrected q< 0.05). Dpb = *D. p. bogotana*, Dpp *= D. p. pseudoobscura*, BC_1p_ = First generation backcross from *D. p. pseudoobscura* females, BC_1b_ = First generation backcross from *D. p. bogotana* females, BC_4_ = Fourth generation backcross, F8^y^ = yellow introgression, F8^y+^ = non-yellow introgression, F8^se^ = sepia introgression, F8^se+^ = non-sepia introgression

	Dpb	Dpp	BC_1p_	BC_1b_	BC_4_	F8^y^	F8^y+^	F8^se^	F8^se+^
**GA13457**	−14.95	−11.66	−8.56*	−7.20*	−9.95	−7.27*	−8.67*	−7.97*	−7.08
**GA14907**	−2.69	−2.30	−1.61	−1.12	−2.94	−1.67	−1.86	−2.00	−1.74
**GA15058**	−12.10	−7.83	−5.99	−5.43*	−6.02	−8.83	−7.68	−8.40	−8.03
**GA15722**	−1.33	−2.46	−1.16	−0.94	−0.80	−0.78	−1.54	−2.33	−0.93
**GA17870**	−4.17	−4.21	−2.18	−2.67	−2.65	−1.49*	−3.27	−3.23	−0.76
**GA18484**	−8.17	−9.54	−6.08	−5.15*	−8.12	−4.93*	−5.91	−6.35	−5.30
**GA18944**	−8.62	−8.67	−5.37*	−5.16*	−7.54	−5.08*	−6.02	−6.26	−5.31
**GA19543**	−7.36	−7.68	−5.14	−4.88	−6.46	−7.00	−5.39	−5.97	−6.83
**GA20504**	−9.06	−7.68	−5.79	−5.85	−5.87	−10.79	−7.19	−6.59	−12.82
**GA21772**	−9.68	−9.62	−6.51	−6.78	−8.73	−9.31	−7.61	−7.03	−7.05
**GA22690**	−8.57	−9.50	−7.79	−7.27	−8.94	−8.79	−7.61	−7.88	−9.62
**GA24206**	−12.58	−10.15	−6.44*	−6.89*	−7.28*	−9.30	−7.55*	−8.31	−4.90
**GA24796**	−4.35	−5.49	−3.74	−3.31	−8.34	−3.76	−4.28	−6.02	−4.70
**GA25574**	−1.31	−0.56	−1.07	−0.20	−2.97	0.17	−1.09	−0.88	−0.93
**GA26803**	−5.01	−3.52	−3.98	−2.65	−2.89	−5.70	−5.36	−2.73	−3.12
**GA27806**	−9.58	−7.21	−3.84*	−2.81*	−4.55*	−4.75*	−3.85*	−6.21	−5.25
**GA28780**	−11.45	−8.13	−4.04*	−3.43*	−4.99*	−5.09*	−3.79*	−4.80*	−13.37
**GA30092**	−2.09	−1.51	−0.91	0.26	−3.03	0.14	−1.64	−1.88	−2.31
**GA30093**	−6.97	−3.96	−2.81	−1.35*	−5.14	−1.32*	−2.78	−2.96	−3.50

**Figure 3:**
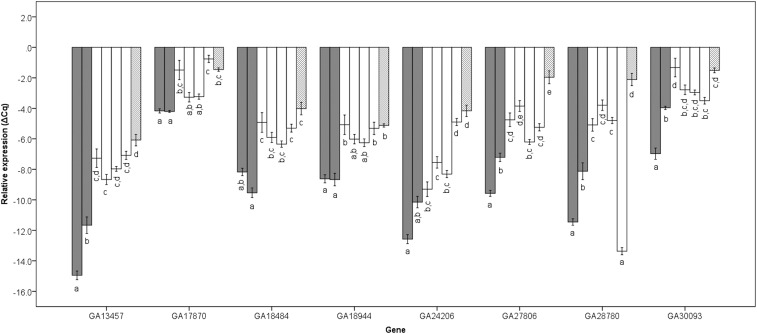
Mean relative expression and standard error of genes misregulated in X-allele introgressions. Parental species are shown in gray, IG progeny in white and sterile F1 males are hatched. For each gene, from left to right, *D. p. bogotana*, *D. p. pseudoobscura*, F8^y^, F8^y+^, F8^se^, F8^se+^. Shared letters identify non-significantly different groups (post-hoc Scheffe’s test, FDR corrected q< 0.05).

**Figure 4:**
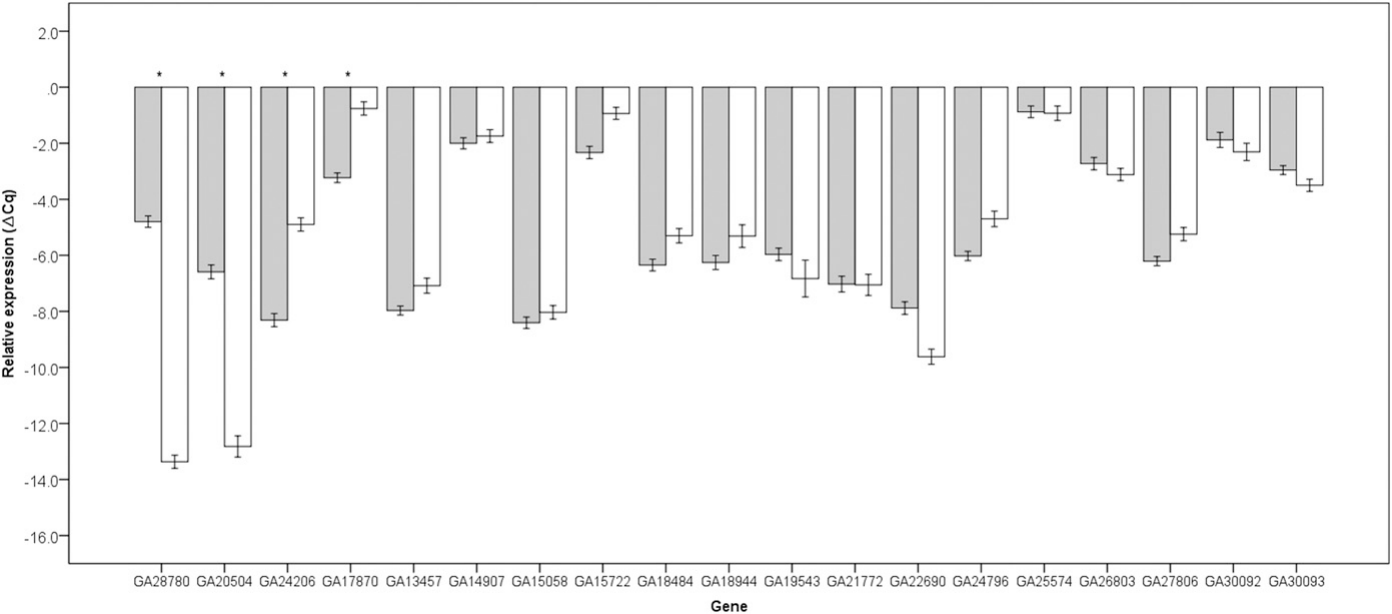
Mean relative expression and standard error of genes in fertile sepia (gray bars) and sterile non-sepia (white bars) IG males. Asteriks identify significant differences in expression (post-hoc Scheffe’s test, FDR corrected q< 0.05).

The analysis of X chromosome allele introgression progeny revealed eight genes (GA13457, GA17870, GA18484, GA18944, GA24206, GA27806, GA28780, and GA30093) misregulated in fertile IG progeny relative to parental species (Scheffe’s post-hoc test; Table 2). Except for GA24206, all these genes had at least one fertile introgression progeny misregulated at levels not significantly different from sterile F1 males (Scheffe’s post-hoc test; Figure 3). The combined results from the fertile BC (Figure 2) and IG males (Figure 3) allows us to exclude nine genes as those whose misregulation in the sterile F1 male hybrid condition could be directly associated with sterility. The analysis of the X^y^ and X^se^ allele introgressions provided evidence in support of proteases misregulated by X-autosomal unbalanced interactions. GA13457, GA18484 and GA18944 were equally overexpressed in introgressions that selected for different regions of the X chromosome (*y* or *se*) while the other genes showed patterns of misregulation that was influenced by the nature of the X chromosome allele introgressed or the region of the X chromosome introgressed. Among this second group, the significant different expression of genes GA17870, GA20504, GA24206 and GA28780 between *se vs*. *se+* allele introgressions (Figure 4) suggests that these genes are likely targets of *Ovd*.

Altogether, the backcross and introgression designs allowed us to establish that nine of our previously identified proteases uniquely misregulated in sterile F1 hybrid males are misregulated due to cyto-nuclear or X-autosomal unbalances, but their misexpression is not directly associated with the onset of HMS.

### Candidate HMS proteases and tissue expression

Ten genes showed expression levels similar to parental species in fertile BC and IG males (Table 2). Only four of the nineteen genes assayed showed significantly higher expression in the male accessory glands than testes (Table S2). Because HMS is characterized by the production of mature but immotile sperm (Prakash 1972; Orr 1989; Snook 1998; Gomes and Civetta 2014), candidate HMS genes could be further prioritized as those with higher expression in the testes, where spermatogenesis takes place, relative to male reproductive tissue that contributes seminal fluids to the ejaculate. Among the ten gene candidates, we found nine genes with higher levels of expression in the testes than the accessory glands and one gene (GA26803) with the reverse pattern (Table 3 and Table S2). Further, GA21772 was very lowly expressed (∆Cq < -10) and GA15722 very highly expressed (∆Cq > -5) in all male reproductive tissue samples (Table S2). Moreover, GA15722 is highly expressed in both males and females reproductive and non-reproductive anatomy (https://flybase.org/) suggesting GA15722 is a housekeeping gene that when disrupted could affect more than just sperm function.

**Table 3:** Mean male reproductive tissue expression for candidate proteases not misregulated in fertile BC/IG progeny. The ratio of expression in testes (sperm) relative to the accessory glands (seminal fluids) and fold change are used to identify genes as testes or accessory gland enriched

	T	AG	Ratio	Fold-change	Tissue enriched
GA14907	−2.03	−17.05	0.12	8.33	Testes
GA15722	−2.09	−4.53	0.46	2.17	Testes
GA19543	−7.44	−11.31	0.65	1.54	Testes
GA20504	−6.47	−19.15	0.34	2.94	Testes
GA21772	−10.28	−17.79	0.58	1.72	Testes
GA22690	−8.62	−19.15	0.45	2.22	Testes
GA24796	−4.24	−15.88	0.27	3.70	Testes
GA25574	0.40	−11.14	0.04	25.00	Testes
GA26803	−12.48	−1.99	6.27	6.27	Accessory Glands
GA30092	−0.63	−11.97	0.05	20.00	Testes

### Limited interspecies sequence differences in putative cis-regulatory regions

We sequenced over 1.5kb for the seven candidate HMS genes with higher expression in testes. The alignment of our sequences to the *D. p. pseudoobscura* gene sequences available in FlyBase are found in the figshare repository, which also shows substitutions identified as shared polymorphisms after BLASTn searches to the Sequence Read Archive (SRA). Table 4 summarizes the location of fixed substitutions between species in relation to the TSS. Overall, we found a very limited number of fixed nucleotide changes. Genes GA30092 and GA22690 had the highest proportion of fixed changes (0.004 and 0.009 respectively). GA22690 was the only gene to show a significant (*P* < 0.05) monotonic increase in substitutions that corresponded to eight changes, from -751 to -525, in the promoter distal region (Table 4).

**Table 4:** Confirmed fixed changes between species after removing shared polymorphisms (see Supplementary material). Position is relative to the transcription start site (TSS). Domains are T = Transcript; CDS = protein coding; *P* = promoter (-200,+200); PP = Promoter proximal (-500); PD = Promoter distal (<-500). NA = not applicable (*i.e.*, no fixed substitutions)

Gene	sequenced	length	Position	Domain	Substitution (pse > bog)
**GA14907**	−874...+674	1,548	NA	NA	NA
**GA19543**	−712...+1,601	2,313	+1,250	CDS	C > T
			+1,251	CDS	T > G
			+1,291	CDS	C > T
**GA20504**	−1,063...+659	1,722	+440	CDS	G > C
			+556	CDS	T > C
**GA22690**	−1,123...+518	1,641	−1052	PD	T > (-)
			−1015	PD	G > A
			−751	PD	A > G
			−711	PD	G > A
			−706	PD	C > G
			−692	PD	A > C
			−626	PD	A > T
			−573	PD	C > A
			−526	PD	T > G
			−525	PD	A > G
			−348	PP	G > T
			−303	PP	G > A
			−289	PP	G > T
			−84	P	A > G
			+232	CDS	C > T
**GA24796**	−1,091...+509	1,600	+447	T(5′UTR)	A > T
			+508	CDS	C > A
**GA25574**	−1,211...+362	1,573	NA	NA	NA
**GA30092**	−1,139...+528	1,710	−1081	GA25574	C > T
			−965	GA25574	T > G
			−728/-725	PD	ATAC > (—-)
			+149	CDS/P	G > T
			+444	CDS	G > C
			+502	CDS	G > T
			+504	CDS	T > A

## Discussion

The genetic basis of reproductive isolation between species is typically addressed within the framework of the Dobzhansky-Muller model (Dobzhansky 1937; Muller 1942) and it has been previously shown that divergence in gene regulation is a major contributor to the evolution of Dobzhansky-Muller incompatibilities between species of *Drosophila* (Haerty and Singh 2006). Yet, we lack specific examples of gene × gene interactions. Moreover, to understand variation in gene expression between species, it is crucial to map specific gene × gene interactions and ultimately gene regulatory networks (Signor and Nuzhdin 2018).

Here, we have mapped gene × gene interactions affecting expression of proteolytic candidates HMS genes by using a genetic approach that generated males genotypically similar to F1 sterile male hybrids but with different (*i.e.*, *D. p. pseudoobscura vs. D. p. bogotana*) alleles. Four genes were differentially expressed based on the introgression of a sepia eye color marker (linked to the major HMS gene *Ovd*). Three of the four genes, GA17870, GA24206 and GA28780, were misregulated in BC or IG fertile males. Thus, the misregulation of these three genes cannot be directly linked to the onset of HMS. Why these genes are modulated by *Ovd* but not directly linked to sterility could also be explained by possible pleiotropic effects of *Ovd*. For example, *Ovd* is known to influence progeny sex-ratio (Phadnis and Orr 2009; Phadnis 2011) and the biological processes in which *Ovd* is involved are still unknown (https://flybase.org/). GA17870 is a male AG gene with very low expression in other MRT tissues. We know that seminal fluids play an important function in postmating reproductive female biology (LaFlamme and Wolfner 2013), but it remains to be determined whether sterile male hybrids between *D. p. bogotana* and *D. p. pseudoobscura* are impaired in terms of their postmating reproductive fitness due to the misregulation of AG genes like GA17870. GA20504 is arguably the most interesting candidate emerging from our study. The gene is differentially expressed based on the introgression of the sepia allele linked to *Ovd*, restores normal expression in fertile BC and IG males but becomes misregulated in introgressed male carriers of the *Ovd* sterility allele. Not much is currently known about GA20504 but one of its possible mammalian, including human, orthologs is the gene alanyl aminopeptidase (ANPEP) (https://flybase.org/). Overexpression of endopeptidase activity in man has been linked to sperm lack of motility and both ANPEP, and Neutral endopeptidase (NEP), are targetable through inhibitors to help restore sperm motility in man (Subirán *et al.* 2010; Bosler *et al.* 2014).

Our previous finding of proteases being the largest class of genes uniquely misregulated in the MRT of sterile F1 hybrid males was intriguing because proteases had not been previously identified as a possible contributor to HMS in *Drosophila*. Here, we show that F1 hybrid male sterile misregulation of nine previously identified proteases can be explained by differences in X-autosomal and nuclear-maternal asymmetries without a direct link to the sterility phenotype. Of the remaining ten candidates, seven can be prioritized as directly linked to the HMS sterility phenotype based on the genes’ site and level of expression within different tissue of the MRT. Except for GA19543, information available on the other six candidate HMS genes allow us to discuss them in the context of the sterility phenotype. GA20504 was discussed above as a gene of interest based on our results and its possible orthology to proteases that have shown associations between changes in gene expression and sperm motility. GA24796 is within a previously mapped third chromosome HMS sterility QTL between *D. p. pseudoobscura* and *D. p. bogotana* (Phadnis 2011; Gomes and Civetta 2015). GA22690 also appears to be located within a previously identified X chromosome HMS QTL based on its genome location (https://flybase.org/), but the precise genome position of the QTL is harder to determined due to the use of an unannotated phenotypic marker (*sd*) in the QTL mapping study (Phadnis 2011). GA14907 is the gene ortholog to a known *D. melanogaster* sperm protein (S-Lap 5), which is upregulated during meiotic and post-meiotic stages of sperm development (Dorus *et al.* 2011). Sperm-LeucylAminoPeptidases have expanded and functionally diversified during *Drosophila* evolution, and S-LAP 5 is one of the highly abundant proteins present in the *D. melanogaster* sperm proteome (Dorus *et al.* 2011). Both *D. melanogaster* S-LAP 5 and *D. pseudoobscura* GA14907 are conserved at a lysine residue (K3390), which is essential for enzymatic activity (Strater *et al.* 1999). GA25574 and GA30092 are located within a 4.5Kb region of the *D. pseudoobscura* second chromosome that also includes another protease we reported as uniquely misregulated in the MRT of F1 sterile hybrids in our prior genome-wide expression study (GA30093) (Gomes and Civetta 2015). The genomic proximity of these proteases, the fact that they are all putative serine-endopeptidase inhibitors and that they share high expression in testes suggests that all three genes are products of gene duplication event/s with possible subfunctionalization. The three genes are orthologs to a single *D. melanogaster* gene (CG42827) whose expression is significantly changed in primary spermatocytes of knockdowns of testes meiotic arrests bromomodomain proteins (tBRDs) (Theofel *et al.* 2014, 2017). The transcription of genes required during spermatid differentiation is regulated by testes meiotic arrest complex (tMAC) proteins and the *D. melanogaster* ortholog of GA25574 and GA30092 is one such spermatid target, lending further support to the role of these two genes in HMS.

Variation in gene expression between species is characterized by a preponderance of *cis* changes that affects the expression of target genes (Wittkopp *et al.* 2008; Emerson and Li 2010; Gordon and Ruvinsky 2012; Coolon *et al.* 2014; Nourmohammad *et al.* 2017; Metzger *et al.* 2017). The sequencing of upstream gene regions for our seven candidate HMS proteases found no evidence, except for GA22690, of an enrichment of substitutions in *cis*. Although we assayed only seven genes, this information suggests that the genome-wide pattern of higher divergence in *cis* than *trans* between species might not necessarily apply to genes whose misregulation is linked to the establishment of reproductive isolation barriers. Because many of the genome-wide *cis*-regulatory changes are often compensated by changes in *trans* (see Signor and Nuzhdin 2018 for a review), it is feasible that changes in only a few *trans* regulatory sequences (*i.e.*, proteins) could be enough to affect the expression of several genes across gene networks thus triggering the onset of reproductive isolation. The general lack of changes in *cis* that we report for candidate HMS proteases also provides guidance regarding any future attempt to map *trans-cis* interactions. For example, our results argue against ChIP assays conducted to identify binding directly upstream of our identified targets.

In summary, we have used genetic backcrossing and introgression combined with analysis of gene expression to narrow down our prior list of proteases uniquely misregulated in sterile F_1_ hybrid males (Gomes and Civetta 2015) to a handful of candidate whose misexpression can be directly associated with HMS. While there have been several studies mapping and quantifying epistatic interactions, only a very limited number of interacting genes associated with incompatibilities have been identified (Brideau *et al.* 2006; Bhattacharyya *et al.* 2014). Here, we have identified four partners of the *Ovd* gene, with GA20504 having a direct association with the sterility phenotype. We have also established that the misregulation of most candidate HMS proteases is not driven by an accumulation of *cis*-regulatory changes. Moving forward, the precise functional role of these candidate genes shall be assessed by the generation of genetically engineered strains in which the activity of the candidate genes is either enhanced or silenced.
